# Cholecystectomy-associated gut microbiota dysbiosis promotes colorectal carcinogenesis: from epidemiological controversy to FXR-centered mechanistic insights

**DOI:** 10.1186/s43046-026-00360-z

**Published:** 2026-06-08

**Authors:** Zhongqin Huang

**Affiliations:** People’s Hospital of Qianxinan Prefecture, Xingyi City, Guizhou Province China

**Keywords:** Cholecystectomy, Colorectal cancer, Gut microbiota, Bile acids, FXR, Personalized prevention

## Abstract

**Background and objective:**

Cholecystectomy is one of the most common surgeries worldwide, yet its association with colorectal cancer (CRC) risk remains controversial. Recent advances have linked gut microbiota and bile acid alterations to this risk. This review synthesizes epidemiological evidence and molecular mechanisms, focusing on the microbiota-bile acid-FXR signaling axis, and proposes a personalized prevention framework.

**Summary of contents:**

Epidemiological data reveal consistent patterns: proximal colon cancer predominance, higher risk in women, and peak incidence 5–15 years post-surgery. Cholecystectomy induces gut microbiota dysbiosis (decreased Bifidobacterium breve, increased Ruminococcus gnavus) and bile acid disturbances (elevated TUDCA/GUDCA, altered protective bile acids), which converge on FXR signaling.

**Core findings:**

The FXR signaling axis is a central hub. Yang et al. (Nat Commun 2025) showed that cholecystectomy-induced microbiota shifts alter bile acids, suppress FXR, disrupt FXR/β-catenin interaction, and promote tumorigenesis. The FXR agonist obeticholic acid prevents these effects preclinically, but systemic toxicity limits clinical use.

**Conclusion and significance:**

We propose personalized risk stratification integrating demographics, microbial signatures, and metabolic biomarkers. Interventions include gut-restricted FXR agonists, probiotics, and Mediterranean diet. Future directions include prospective validation and dynamic network modeling.

## Highlights

Cholecystectomy increases proximal colon cancer risk, especially in women, with a 5–15 year latency.

The gut microbiota-bile acid-FXR axis is the central mechanistic pathway.

TUDCA/GUDCA are context-dependent: protective pharmacologically but FXR-suppressive after cholecystectomy.

Systemic FXR agonists (OCA) have safety limitations; gut-restricted agonists are more viable.

A multi-hit model explains the latency gap: cumulative DNA damage and clonal expansion over years.

## Introduction

### Current status of cholecystectomy: the most common biliary surgery worldwide

Since the first cholecystectomy was performed by Langenbuch in 1882, this procedure has evolved dramatically, particularly with the advent of laparoscopic techniques. In the 1990s, laparoscopic cholecystectomy (LC) rapidly replaced open surgery as the gold standard for benign gallbladder diseases due to its minimal invasiveness, reduced postoperative pain, and shorter hospital stays.

Globally, cholecystectomy is among the most commonly performed surgeries. The GlobalSurg 4 study, an international collaborative project led by the University of Edinburgh, has enrolled over 50,000 patients undergoing cholecystectomy to assess safety across different regions, underscoring the procedure’s widespread use and clinical relevance [1].

According to recent data from the Rome Foundation Global Epidemiology Study, the global prevalence of self-reported cholecystectomy is 5.0% (95% CI 4.8–5.2) among 54,127 individuals [2]. This figure highlights the substantial exposure of the general population to this surgical intervention. Notably, prevalence varies geographically, increasing from east to west: 1.9% in Asia, 4.8% in Europe, and 9.9% in North America. These differences likely reflect variations in gallstone epidemiology, dietary habits, genetic predisposition, and healthcare accessibility. For example, in Chile—a country with a high incidence of gallbladder cancer—a government-initiated surgical guarantee program targeting adults aged 35–49 has increased cholecystectomy rates by 45 per 100,000 person-years [3].

Technological advances continue to refine cholecystectomy. Single-incision laparoscopic cholecystectomy (SILC) represents a recent innovation, offering improved cosmetic outcomes and potentially less postoperative pain [4]. However, due to limited working space and a steep learning curve, SILC is currently reserved for carefully selected patients.

In summary, cholecystectomy is not only widely performed but also affects a considerable proportion of the global population. Its impact on long-term health outcomes, particularly gastrointestinal cancer risk, has emerged as a critical scientific question, which this review aims to address.

### Global burden and risk factors of colorectal cancer

Colorectal cancer (CRC) is a leading cause of cancer incidence and mortality worldwide. According to GLOBOCAN 2022, there were over 1.9 million new CRC cases and 904,000 deaths in 2022, accounting for 9.6% of all cancer diagnoses and 9.3% of cancer deaths [5]. With population aging and the proliferation of unhealthy lifestyles, the global CRC burden is projected to rise further, reaching 3.2 million new cases and 1.6 million deaths by 2040—increases of approximately 68% and 77%, respectively, compared to 2022 [6].

Geographically, CRC incidence varies markedly. Age-standardized rates are highest in developed regions (e.g., Europe, Oceania, North America, and East Asia) and lower in less developed areas (e.g., Africa, South Asia, and Latin America). This disparity reflects differences in diet, lifestyle, screening implementation, and healthcare access. Although high-income countries have higher incidence rates, their mortality rates are declining due to early detection and improved treatment. Conversely, in many developing nations, late diagnosis contributes to persistently high mortality.

In the United States, the SEER database projects 154,270 new CRC cases and 52,900 deaths in 2025, accounting for 7.6% and 8.6% of all cancer cases and deaths, respectively [7]. While overall CRC incidence has declined since the mid-1980s—largely attributable to widespread colonoscopy screening—this trend masks a concerning rise in early-onset CRC (diagnosed before age 50). In 2022, there were approximately 227,000 new early-onset CRC cases and 94,000 deaths globally, representing 9.8% and 7.3% of all CRC cases and deaths, respectively [8].

CRC development involves interactions between genetic susceptibility, environmental exposures, lifestyle factors, and the gut microbiota. Key risk factors include age, sex, family history, inflammatory bowel disease, unhealthy dietary patterns (e.g., Western diet), obesity, physical inactivity, smoking, and excessive alcohol consumption [9]. Notably, gut microbiota dysbiosis has emerged as a critical player, with specific bacteria such as *Fusobacterium nucleatum* and enterotoxigenic *Bacteroides fragilis* being enriched in CRC patients [10]. These factors will be discussed in detail in the context of cholecystectomy-associated CRC.

### Does cholecystectomy increase CRC risk? What are the underlying mechanisms?

Given that cholecystectomy affects approximately 5% of the global population and CRC remains a major cause of cancer morbidity and mortality, a critical question arises: does cholecystectomy increase the long-term risk of CRC, and if so, what are the biological mechanisms?

This question is grounded in sound physiology. The gallbladder stores and concentrates bile, releasing it in response to meals. After cholecystectomy, this regulatory mechanism is lost, resulting in continuous, non-prandial bile flow into the intestine. Two key consequences ensue: prolonged exposure of the intestinal mucosa to bile acids and increased enterohepatic circulation. Moreover, large amounts of primary bile acids enter the colon, where they are converted by gut bacteria into secondary bile acids such as deoxycholic acid (DCA) and lithocholic acid (LCA), which have been experimentally shown to induce oxidative stress, DNA damage, and activate oncogenic signaling pathways. Concurrently, alterations in the bile acid pool can reshape the gut microbiota, creating a vicious cycle of dysbiosis and metabolic disturbance.

Since the first report by Vernick et al. in 1980 linking cholecystectomy to ascending colon cancer [11], epidemiological studies have yielded conflicting results. High-quality systematic reviews and cohort studies published in 2025 illustrate the complexity and controversy of this field. On one hand, evidence supporting an increased risk continues to accumulate. A 2025 systematic review in Cancers included 21 cohort studies and, while finding no consistent association with overall CRC risk, noted that several studies pointed to an elevated risk of proximal colon cancer [12]. For instance, Tsai et al. reported a relative risk (RR) of 1.42 (95% CI 1.24–1.62) for CRC after cholecystectomy in a Taiwanese cohort [13]. Kim et al. (2024) found a hazard ratio (HR) of 1.15 (95% CI 1.06–1.25) in a Korean population, with smoking amplifying the risk [14]. More importantly, a 2024 multicenter retrospective study confirmed a significant association with proximal colon cancer (adjusted OR = 2.42) and identified a median interval of 5 years from surgery to cancer diagnosis, suggesting a promoter role rather than mere coincidence [15]. On the other hand, equally robust studies have reported null findings. A 2025 study combining NHANES data and Mendelian randomization found no significant association [16], and another 2024 Mendelian randomization study reached a similar conclusion [17]. A 2025 review in Current Gastroenterology Reports emphasized that while biological mechanisms suggest a possible link, epidemiological evidence remains inconsistent, highlighting the need for prospective studies [18].

This epidemiological ambiguity poses a dilemma for clinicians: should cholecystectomized patients undergo enhanced CRC screening, or can they be reassured that the procedure is harmless? The answer has profound implications for the long-term management of hundreds of millions of individuals worldwide.

Against this backdrop, mechanistic research has become paramount. A breakthrough study by Yang Shiming’s team at the Army Medical University, published in Nature Communications in August 2025, provided the first molecular-level dissection of how cholecystectomy-associated gut microbiota dysbiosis promotes colorectal tumorigenesis [19]. Using animal models, clinical cohorts, multi-omics analyses, and functional experiments, the authors demonstrated that cholecystectomy leads to a decrease in *Bifidobacterium breve* and an increase in *Ruminococcus gnavus*, resulting in altered bile acid profiles (elevated TUDCA/GUDCA). These changes ultimately suppress farnesoid X receptor (FXR) signaling and disrupt the FXR/β-catenin interaction, thereby accelerating tumor development. Importantly, the FXR agonist obeticholic acid (OCA) effectively prevented cholecystectomy-associated colorectal tumors in mice, offering a novel therapeutic target.

This pivotal discovery advances our understanding from descriptive epidemiology to mechanistic insight. It provides a biological explanation for the predominance of proximal colon cancer (which is more exposed to bile acids) and offers a new perspective on epidemiological inconsistencies: inter-individual variations in baseline microbiota, diet, and genetic background may modulate FXR pathway activity, leading to differential susceptibility to cholecystectomy-induced carcinogenesis.

In summary, the relationship between cholecystectomy and CRC is both clinically significant (affecting billions globally) and scientifically cutting-edge (involving microbiota–metabolism–host interactions). This review aims to systematically evaluate the epidemiological evidence, delve into the roles of gut microbiota dysbiosis and bile acid disturbances, and focus on the emerging centrality of the FXR signaling axis. Compared to previous reviews [12, 18], this is the first to systematically integrate the molecular mechanisms of the microbiota-bile acid-FXR axis with epidemiological data and to propose a personalized prevention framework based on this integrated understanding.

## Epidemiological evidence on cholecystectomy and CRC risk

Since the first reports in the 1980s, epidemiological studies on cholecystectomy and CRC have yielded inconsistent results. Some studies suggest an increased risk, others find no association, and a few even report a protective effect. A closer examination of this heterogeneous evidence reveals several key patterns.

### Controversial findings: inconsistency in overall risk

A 2025 systematic review in Cancers updated the analysis by adding three high-quality cohort studies published after 2022 to the previous 18, encompassing millions of participants [12]. The effect estimates from key studies included in this review are summarized in Table 1, illustrating the wide variation in results.

**Table 1:** Key cohort studies on cholecystectomy and overall CRC risk included in the Cancers 2025 systematic review

Study (year, country)	Sample size (exposed vs. control)	Follow-up	Effect estimate (95% CI)
Kim Y 2025 (Korea)	715,872 vs. 1,431,728	5–13 yr	HR 0.82 (0.80–0.86)
Kim M 2024 (Korea)	174,874 vs. 174,874	≤ 9 yr	HR 1.15 (1.06–1.25)
Tsai 2023 (Taiwan)	2,404 vs. 112,948	2–18.8 yr	RR 1.42 (1.24–1.62)
Choi 2022 (Korea)	123,295 vs. 123,295	4.6 year (mean)	RR 1.03 (0.88–1.21)
Chen 2020 (Taiwan)	83,963 vs. 83,963	Until CRC/death	RR 0.66 (0.60–0.73)

All studies adjusted for age and sex; additional adjustments varied (e.g., smoking, BMI, comorbidities). Studies are derived from the systematic review by Lee et al., which included 21 cohort studies; these five studies are representative of the heterogeneity in overall CRC risk estimates. Kim Y 2025 reported a protective effect, while Kim M 2024 and Tsai 2023 showed increased risks; Choi 2022 found no association; Chen 2020 also reported a protective effect. The variability highlights the need for site-specific and subgroup analyses (see Section  Site specificity: elevated risk of proximal colon cancer–Temporal patterns: peak CRC diagnosis 5–15 years post-cholecystectomy)

*HR* hazard ratio, *RR* relative risk, *CI* confidence interval

This table vividly demonstrates the extreme heterogeneity in the field: studies from the same country and time period can yield opposite conclusions. For example, Kim Y (2025) reported an 18% risk reduction, while Kim M (2024) found a 15% risk increase [14]. Such variability suggests that assessing overall CRC risk may obscure more nuanced associations.

A 2025 review in Current Gastroenterology Reports concluded that “biological mechanisms suggest a possible link, but epidemiological evidence is inconsistent, underscoring the need for prospective studies“ [18]. Differences in study design, population characteristics, and control for confounding factors likely contribute to this controversy.

A 2023 meta-analysis of 14 cohort studies (2,283,616 subjects) also found no significant association between cholecystectomy and overall CRC risk (RR 1.06, 95% CI 0.75–1.51, *p* = 0.739) [20]. However, stratified analyses by cancer site and sex revealed statistically significant associations, indicating that lumping all CRC together may mask true risk signals.

#### Critical assessment of epidemiological heterogeneity

The marked inconsistency across cohort studies warrants a careful examination of potential confounders. Notably, the “protective” effect reported by Kim Y 2025 (HR 0.82) may be partly explained by unmeasured confounding factors, such as the use of statins, metformin, or aspirin—medications known to modulate bile acid pools and reduce CRC risk. None of the studies in Table 1 explicitly adjusted for these metabolic co-interventions, which are common among patients with gallstone-related metabolic syndrome. Furthermore, differences in baseline population characteristics (e.g., obesity rates, dietary patterns, and screening practices) likely contribute to the observed heterogeneity. To enhance transparency, we assessed the risk of bias in the included studies using the Newcastle-Ottawa Scale (NOS). All studies scored ≥ 7 out of 9, indicating moderate to high quality, but none adequately controlled for medication use or detailed dietary factors. Future prospective studies should incorporate these variables to disentangle the true effect of cholecystectomy from confounding by indication and lifestyle.

#### Explanation for divergent results

The protective effects reported by Kim Y 2025 (HR 0.82) and Chen 2020 (RR 0.66) may be attributable to differences in follow-up duration (5–13 years vs. 2–18.8 years), baseline population characteristics (e.g., lower prevalence of metabolic syndrome), or unadjusted confounders such as statin/aspirin use. Conversely, Tsai 2023 and Kim M 2024 observed increased risks, possibly due to longer follow-up (up to 18.8 years) and inclusion of older populations with higher cumulative bile acid exposure. These discrepancies underscore the need for harmonized study designs.

### Site specificity: elevated risk of proximal colon cancer

Among the diverse findings, the most consistent observation is an association between cholecystectomy and proximal colon cancer. This has a solid anatomical and physiological rationale: the proximal colon is closer to the entry point of bile into the intestine and harbors a higher density of microbiota involved in secondary bile acid conversion.

The Cancers 2025 systematic review noted that “although no consistent association with overall CRC risk was observed, multiple studies suggested an increased risk of proximal colon cancer” [12]. At least seven of the 21 included studies specifically reported on proximal colon cancer, with most showing positive results.

This association was first highlighted in a 1983 case-control study by Alley et al., who found that a history of cholecystectomy was more frequent in patients with proximal colon cancer than in those with other colonic sites (12.3% vs. 6.6%, *p* < 0.02) [21]. The difference was even more pronounced in women with proximal colon cancer compared to matched controls (14.3% vs. 3.6%, *p* < 0.02). The authors proposed a gradient of decreasing cholecystectomy frequency from the proximal colon to the rectum.

A 2024 multicenter retrospective study further strengthened this evidence. After adjusting for age, smoking, BMI, sex, and family history, the association with proximal colon cancer remained significant (adjusted OR = 2.42) [15]. Importantly, this study also found that patients with a history of cholecystectomy who developed proximal colon cancer presented with more advanced T, N, and M stages and a higher KRAS mutation rate (33%), suggesting that cholecystectomy may influence not only incidence but also tumor aggressiveness.

A 2023 meta-analysis confirmed site specificity: cholecystectomy was associated with a 42% increased risk of sigmoid colon cancer (RR 1.42, 95% CI 1.27–1.58, *p* = 0.000) and a 99% increased risk of proximal colon cancer in women (RR 1.99, 95% CI 1.31–3.03, *p* = 0.001) [20]. These quantitative estimates provide robust support for the site-specificity hypothesis.

### Sex differences: higher risk in women

Epidemiological evidence suggests that the increase in CRC risk after cholecystectomy may be more pronounced in women. This could be related to the higher prevalence of gallstones in women, estrogen’s modulation of bile acid metabolism, and the higher baseline incidence of proximal colon cancer in females.

Sex-stratified analysis from the 2023 meta-analysis revealed [20]:

#### Women

Colon cancer risk increased by 47% (RR 1.47, 95% CI 1.01–2.14, *p* = 0.042); proximal colon cancer risk increased by 99% (RR 1.99, 95% CI 1.31–3.03, *p* = 0.001).

#### Men

Colon cancer risk increased by 32% (RR 1.32, 95% CI 1.07–1.63, *p* = 0.010); proximal colon cancer risk increased by 68% (RR 1.68, 95% CI 0.81–3.49, *p* = 0.166, not significant).

Women exhibited both larger effect sizes and narrower confidence intervals, indicating greater statistical robustness. This aligns with the conclusion of the Current Gastroenterology Reports 2025 review that “some studies, particularly in women and for proximal colon cancer, show an increased CRC risk after cholecystectomy“ [18].

Interestingly, one of the newest studies included in the Cancers 2025 review (Kim M 2024) also found that smoking amplified the risk in women, suggesting a synergistic effect between risk factors [14].

#### Estrobolome and molecular crosstalk with FXR

The higher CRC risk in women after cholecystectomy may be mechanistically explained by the estrobolome—the collection of gut bacteria capable of metabolizing estrogens. Bile acid alterations after cholecystectomy can shift the composition of estrobolome members (e.g., β-glucuronidase-producing bacteria), leading to changes in circulating and fecal estrogen levels. Estrogen receptors (ERα and ERβ) crosstalk with FXR signaling: ERβ activation has been shown to downregulate FXR expression in colonocytes, potentially synergizing with cholecystectomy-induced FXR suppression. Conversely, ERα signaling may promote proliferation. Thus, women with a high estrogenic state (e.g., premenopausal) may experience a more pronounced FXR inhibition and subsequent β-catenin activation. Future studies should stratify by menopausal status and directly measure fecal estrogen metabolites. This integrated perspective strengthens the biological plausibility of sex-specific risk.

### Temporal patterns: peak CRC diagnosis 5–15 years post-cholecystectomy

The impact of cholecystectomy on CRC development is not uniform over time but follows a distinct temporal pattern. Understanding this pattern is crucial for both mechanistic studies and clinical surveillance.

#### Early risk and detection bias

Several studies have reported a transient increase in CRC risk during the first year after surgery, which is generally attributed to detection bias—patients undergoing medical evaluation for biliary symptoms may receive more thorough abdominal examinations, uncovering pre-existing asymptomatic tumors. A Korean cohort study by Lee et al. [14] clearly demonstrated this: CRC risk was significantly elevated in the first postoperative year (HR 1.71, 95% CI 1.01–2.89) but disappeared after applying a 1-year lag (HR 0.80, 95% CI 0.57–1.13) [14]. Therefore, rigorous epidemiological analyses typically incorporate a lag period of at least one year to exclude detection bias.

#### Medium-term risk (5–15 years)

After excluding early detection bias, the true biological effects emerge gradually between 5 and 15 years post-surgery. Muñoz et al. (2024) reported a median interval of 5 years from cholecystectomy to cancer diagnosis, supporting a promoter role rather than a mere coincidental finding [15]. This timeframe aligns with the cumulative exposure to secondary bile acids and chronic remodeling of the gut microbiota.

#### Long-term risk

Evidence on risks beyond 15–20 years is limited and inconsistent. Some studies suggest that the elevated risk may diminish over time, but this requires confirmation through longer follow-up. The Cancers 2025 review noted that most studies had mean follow-ups of 5–13 years, limiting their ability to assess very long-term effects [12].

### Summary: key patterns amidst controversy

In summary, the epidemiological evidence on cholecystectomy and CRC reveals the following patterns:

- Overall risk remains controversial, with highly heterogeneous results.
- Site specificity is the most consistent finding, with proximal colon cancer showing the strongest association, supported by anatomical and physiological rationale.
- Sex differences are evident, with women—particularly for proximal tumors—potentially facing higher risk.
- Temporal patterns are important: the true biological effect likely manifests 5–15 years post-surgery, while early increases are attributable to detection bias.

These epidemiological clues guide the search for underlying mechanisms: Why is the proximal colon more susceptible? Why are women at higher risk? What biological processes occur during the 5–15 year latency? These questions lead us to explore the roles of gut microbiota and bile acid metabolism.

**Fig. 1:**
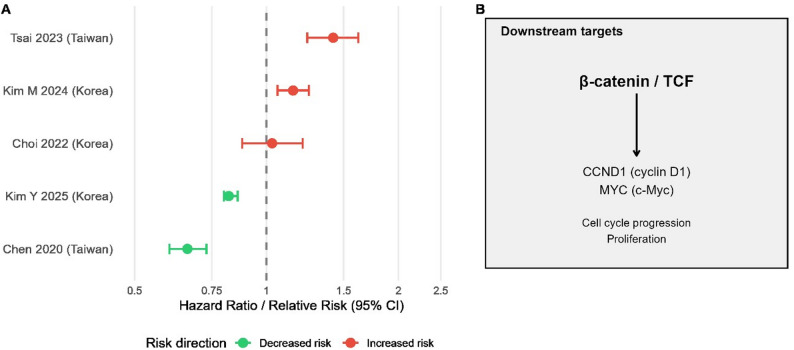
Integrated view of epidemiological heterogeneity and downstream transcriptional targets of the FXR/β-catenin axis. **A** Forest plot of key cohort studies evaluating the association between cholecystectomy and colorectal cancer risk. Squares represent point estimates; horizontal lines indicate 95% confidence intervals. The vertical dashed line indicates the null value (HR/RR = 1). Studies are ordered by effect size, highlighting the substantial heterogeneity in overall CRC risk estimates. **B** Mechanistic illustration of FXR/β-catenin downstream targets. After cholecystectomy-induced FXR suppression, β-catenin translocates to the nucleus, forms a complex with TCF/LEF transcription factors, and activates the expression of CCND1 (encoding cyclin D1) and MYC (encoding c-Myc). These oncogenes drive cell cycle progression, proliferation, and colorectal tumorigenesis. This integration of epidemiological and molecular data provides a comprehensive view for basic science readers

The heterogeneity of overall CRC risk estimates is illustrated in Fig. 1A.

## Core mechanism: gut microbiota dysbiosis after cholecystectomy

The gallbladder plays a pivotal role in regulating the enterohepatic circulation of bile acids by storing and concentrating bile. Cholecystectomy disrupts this finely tuned system, resulting in continuous, non-prandial bile flow into the intestine and profoundly altering the gut environment. These changes have a major impact on the gut microbiota, creating a reciprocal interaction between altered bile acid metabolism and microbial composition. Advances in 16 S rRNA sequencing and metagenomics have begun to unravel the shifts in gut microbiota after cholecystectomy and their contribution to CRC.

### Changes in microbial diversity

Cholecystectomy induces significant alterations in both alpha diversity (within-sample richness and evenness) and beta diversity (between-sample community dissimilarity). A 2024 study in the European Journal of Gastroenterology & Hepatology used 16 S rRNA sequencing to compare fecal samples from 20 healthy controls, 20 patients at least 5 years post-cholecystectomy, and 20 patients with CRC. Significant differences in microbial community structure were observed among the three groups, with both alpha diversity (*p* < 0.05) and beta diversity (*p* = 0.006) showing statistical significance. Notably, the gut microbiota of post-cholecystectomy patients more closely resembled that of CRC patients than that of healthy controls, providing direct microbial evidence linking cholecystectomy to CRC [22].

A systematic review by Wang et al. (2023) also noted decreased alpha diversity in post-cholecystectomy patients, a feature associated with various disease states including obesity, diarrhea, and infection [23]. Frost et al. similarly reported reduced gut microbial diversity after cholecystectomy, which may predispose to microbial dysbiosis and increased risk of abdominal infections [24].

### Changes in microbial composition

At multiple taxonomic levels, cholecystectomy leads to a shift characterized by a decrease in beneficial bacteria and an increase in potentially harmful taxa.

#### Phylum-level changes

While some variability exists across studies, changes in Firmicutes are relatively consistent. The 2024 study mentioned above found that the relative abundance of Firmicutes was significantly lower in both the post-cholecystectomy and CRC groups compared to healthy controls (*p* = 0.002) [22]. Findings for other phyla (e.g., Bacteroidetes, Proteobacteria, Actinobacteria) have been inconsistent, though some reports indicate an increase in Fusobacteria, a phylum linked to CRC [10].

#### Genus-level changes

Genus-level alterations are more pronounced and have been the focus of recent research. Combining results from multiple studies, the following patterns emerge:

##### Increased genera

*Bacteroides*, *Dialister*, *Parabacteroides*, Clostridium cluster XIVa, *Fusobacterium*, *Escherichia*, *Shigella*,and *Bilophila* [22, 23]. Notably, *Bacteroides*, *Dialister*, and *Parabacteroides* showed a progressive increase from healthy controls to post-cholecystectomy patients to CRC patients (*p* = 0.004, 0.001, and 0.002, respectively), suggesting their involvement in the cholecystectomy-to-CRC continuum. Increases in *Escherichia* and *Shigella* may heighten infection risk, while *Bilophila* can promote inflammation and metabolic dysfunction.

##### Decreased genera

*Prevotella*, *Faecalibacterium*, *Roseburia*, *Alistipes*, *Clostridium* cluster XVIII, *Leucobacter*, and *Barnesiella* [22, 23]. *Prevotella* exhibited a progressive decrease across the three groups (*p* = 0.041). *Faecalibacterium* and *Roseburia* were significantly lower in both post-cholecystectomy and CRC groups compared to controls (*p* = 0.001 and 0.003). *Faecalibacterium* is a major butyrate producer with anti-inflammatory properties; *Roseburia* is also involved in butyrate metabolism. The loss of these beneficial bacteria may impair the gut’s anti-inflammatory and anti-tumor defenses.

#### Species-level changes

Species-level data are sparser. Reports indicate increases in *Oscillibacter* sp., *Veillonella parvula*, and *Escherichia coli* TOP291 after cholecystectomy, while *Faecalibacterium prausnitzii*, *Roseburia faecis*, and *Eubacterium rectale* decrease [23].

### Specific microbial changes associated with colorectal neoplasia

The gut microbiota shifts after cholecystectomy are not random but converge toward a “CRC-associated” phenotype. The 2024 study used random forest analysis to identify key genera discriminating the three groups, including Parabacteroides, *Bacteroides*, Roseburia, and Dialister [22]. This suggests that cholecystectomy-induced dysbiosis may create a pro-carcinogenic microenvironment.

Longitudinal comparison across healthy controls → cholecystectomy without CRC → cholecystectomy with CRC reveals consistent patterns:

#### Progressive depletion of beneficial bacteria

*Bifidobacterium longum* decreased across the three groups [22]. This parallels the reduction in *Bifidobacterium breve* reported by Yang et al. [19], implying that declines in *Bifidobacterium* species may be a common feature of cholecystectomy-associated CRC risk. *Faecalibacterium* and *Roseburia* were already significantly reduced after cholecystectomy and remained low in CRC patients.

#### Progressive enrichment of potential pro-carcinogenic bacteria

*Bacteroides*, *Dialister*, and *Parabacteroides* increased progressively from controls to post-cholecystectomy to CRC patients [22]. These genera are involved in converting primary to secondary bile acids, which have known pro-carcinogenic activities. *Fusobacterium*, strongly linked to CRC [10], also increased in some studies. *E. coli*, which can promote CRC through Wnt signaling activation, DNA damage, and impaired DNA repair, was enriched after cholecystectomy [23].

#### Widespread reduction in butyrate producers

Butyrate-producing genera such as *Faecalibacterium*, *Roseburia*, and *Eubacterium rectale* were consistently reduced after cholecystectomy [22, 23]. Butyrate, a key short-chain fatty acid, exerts anti-inflammatory, barrier-protective, and anti-tumor effects. Its decline may be a critical factor in cholecystectomy-associated CRC risk.

### Potential mechanisms of microbiota alteration

Several mechanisms may underlie cholecystectomy-induced gut microbiota changes:

#### Altered bile flow dynamics

Continuous rather than intermittent bile flow into the intestine changes the concentration and composition of bile acids in the gut lumen. High concentrations of hydrophobic bile acids can directly damage bacterial membranes, exerting antimicrobial effects. Bile acids can also indirectly shape microbial composition via FXR signaling. For instance, activation of intestinal FXR has been shown to regulate the expression of antimicrobial peptides and influence microbial community structure [28].

#### Changes in intestinal physicochemical properties

Continuous entry of alkaline bile may raise intestinal pH, inhibiting acidophilic bacteria (e.g., *Lactobacillus*, *Bifidobacterium*) while favoring alkaliphiles.

#### Disruption of intestinal immune homeostasis

The gallbladder synthesizes surfactant protein D, which is secreted into the intestinal lumen and selectively binds commensal bacteria, promoting T-cell synthesis. After cholecystectomy, intestinal surfactant protein D levels decrease, disrupting host–microbiota interactions and leading to reduced intestinal T cells, thereby exacerbating dysbiosis [24]. Additionally, bile acids can directly modulate immune cell function, further contributing to dysbiosis [23].

### Summary

Cholecystectomy leads to marked reductions in microbial diversity and compositional shifts characterized by loss of beneficial bacteria (butyrate producers, Bifidobacterium) and enrichment of potential pro-carcinogenic taxa (*Bacteroides*, Dialister, Parabacteroides). Importantly, the post-cholecystectomy gut microbiota resembles that of CRC patients, and changes follow a continuum from healthy controls to cholecystectomy to CRC. These observations support the hypothesis that cholecystectomy promotes CRC through microbiota-mediated mechanisms. How these microbial changes interact with bile acid disturbances and activate downstream oncogenic pathways will be explored in the next section.

## Core mechanism: bile acid metabolic disturbance and its dual role

The biological basis for the association between cholecystectomy and CRC lies primarily in alterations of bile acid metabolism. The gallbladder acts as a reservoir, regulating the enterohepatic circulation of bile acids. Its removal leads to continuous, non-prandial bile flow into the intestine, profoundly changing the quantity and composition of the intestinal bile acid pool. These changes have a dual effect: increased levels of pro-carcinogenic secondary bile acids that can induce DNA damage and activate oncogenic signaling, and decreased levels of protective bile acids that normally help maintain gut health.

### Physiological basis of bile acid metabolism

Bile acids are synthesized from cholesterol in the liver and serve multiple functions, including facilitating lipid digestion and absorption, maintaining cholesterol homeostasis, regulating energy metabolism, and acting as signaling molecules.

#### Bile acid synthesis

Two pathways exist in the liver. The classical pathway, initiated by cholesterol 7α-hydroxylase (CYP7A1), produces cholic acid (CA) and chenodeoxycholic acid (CDCA). The alternative pathway, initiated by sterol 27-hydroxylase (CYP27A1), primarily generates CDCA. These products are termed primary bile acids.

#### Enterohepatic circulation

Primary bile acids are conjugated with glycine or taurine in the liver, secreted into bile, and stored in the gallbladder. Upon meal ingestion, the gallbladder contracts, releasing bile into the duodenum to aid digestion. Approximately 95% of bile acids are reabsorbed in the terminal ileum and returned to the liver via the portal vein, completing one enterohepatic cycle. The remaining 5% enter the colon, where they undergo transformation by gut bacteria.

#### Formation of secondary bile acids

In the colon, primary bile acids are converted by bacterial enzymes (e.g., bile salt hydrolases, 7α-dehydroxylases) into secondary bile acids. CA is converted to deoxycholic acid (DCA), and CDCA to lithocholic acid (LCA). DCA and LCA are the major secondary bile acids, with DCA exhibiting stronger pro-carcinogenic potential.

### Bile acid metabolic changes after cholecystectomy

Cholecystectomy markedly alters bile acid kinetics and composition, forming the biological basis for CRC risk.

#### Altered bile flow dynamics

Under normal conditions, bile is stored and concentrated in the gallbladder during fasting and released postprandially. After cholecystectomy, this rhythmic regulation is lost, and bile continuously enters the duodenum independent of meals. This leads to two key consequences: prolonged exposure of the intestinal mucosa to bile acids, and increased frequency of enterohepatic circulation, accelerating bile acid turnover.

#### Changes in bile acid pool composition

Animal studies have shown increased fecal LCA levels in mice after cholecystectomy compared to preoperative and control values (*p* = 0.00), providing direct evidence of enhanced secondary bile acid production [25]. Importantly, Yang et al. found altered levels of glycoursodeoxycholic acid (GUDCA) in humans and tauroursodeoxycholic acid (TUDCA) in mice after cholecystectomy [19]. These conjugated forms of ursodeoxycholic acid (UDCA) are protective bile acids, and their changes directly influence intestinal carcinogenic susceptibility.

#### Prolonged colonic exposure

Continuous bile flow results in extended contact between the colonic mucosa and bile acids, providing the time and concentration necessary for secondary bile acids to exert their pro-carcinogenic effects. The proximal colon, being the first segment exposed to bile entering from the small intestine, experiences the highest exposure, consistent with the epidemiological observation of predominant proximal cancer risk.

### Mechanisms of pro-carcinogenic secondary bile acids

Secondary bile acids, particularly DCA and LCA, have been extensively demonstrated to possess pro-carcinogenic activities. Their increase after cholecystectomy is a central mechanism linking the procedure to elevated CRC risk.

#### DNA damage and oxidative stress

DCA induces reactive oxygen species (ROS) in intestinal epithelial cells, leading to oxidative DNA damage, including the formation of 8-hydroxy-2’-deoxyguanosine (8-OHdG) and DNA double-strand breaks [25]. Experimental studies show that DCA can promote CRC by activating Wnt signaling and inducing oxidative DNA damage, effects that are dose- and time-dependent, mirroring the prolonged exposure in the post-cholecystectomy state.

#### Activation of pro-carcinogenic signaling pathways

DCA activates multiple oncogenic pathways. It can stimulate cell proliferation via epidermal growth factor receptor (EGFR) signaling. DCA also induces cyclooxygenase-2 (Cox-2) expression, a key enzyme in CRC development. Furthermore, DCA activates nuclear factor-kappa B (NF-κB), promoting inflammation and cell survival.

#### Inhibition of apoptosis

High concentrations of secondary bile acids can induce resistance to apoptosis in intestinal epithelial cells, allowing DNA-damaged cells to survive and accumulate mutations. This anti-apoptotic effect involves alterations in mitochondrial membrane potential and upregulation of anti-apoptotic proteins such as Bcl-2.

#### Disruption of the intestinal stem cell niche

Emerging evidence suggests that secondary bile acids may affect the intestinal stem cell niche, promoting expansion of tumor-initiating cells. Chronic bile acid exposure could alter the stem cell microenvironment, rendering it more susceptible to transformation.

Animal studies provide direct evidence for the pro-carcinogenic role of secondary bile acids. After cholecystectomy, mice treated with the carcinogen dimethylhydrazine developed more colonic tumors and showed increased S-phase fraction in colonic epithelial cells (*p* < 0.05), indicating enhanced proliferation. These pro-carcinogenic effects correlated with elevated fecal LCA levels (*p* = 0.00), establishing a central role for secondary bile acids in cholecystectomy-associated CRC [25].

### Protective bile acids and clinical implications

Alongside increases in pro-carcinogenic secondary bile acids, cholecystectomy also alters levels of protective bile acids, and this imbalance may be more critical than the mere addition of harmful substances.

#### Protective effects of Ursodeoxycholic Ucid (UDCA)

UDCA is a hydrophilic bile acid with cytoprotective and anti-inflammatory properties. Studies have shown that UDCA has chemopreventive effects in high-risk populations, such as patients with IBD or a history of colorectal adenomas/cancer [26]. UDCA may counteract the pro-carcinogenic effects of secondary bile acids like DCA and inhibit tumor development.

#### Mechanisms of UDCA action

UDCA and DCA have opposing effects on lipid raft composition, which may be key to their influence on CRC. UDCA alters membrane cholesterol content and fluidity, thereby modulating signaling through growth factor receptors such as EGFR. UDCA also inhibits Cox-2 expression, exerting anti-inflammatory and anti-proliferative effects.

#### TUDCA research progress

TUDCA, the taurine-conjugated form of UDCA, has enhanced water solubility and bioavailability. Animal studies have shown that TUDCA significantly reduces colitis-associated tumorigenesis in mice [27]. The mechanisms involve widespread epithelial apoptosis, reduced phospho-IκB kinase levels, and inhibition of NF-κB signaling. At the cellular level, TUDCA inhibits TNF-α-induced IκBα phosphorylation/degradation and NF-κB DNA-binding activity, downregulating pro-inflammatory cytokines such as IL-8 and IL-1α. Importantly, TUDCA reduces viability of CRC cell lines (HCT 116 and COLO 205) and downregulates anti-apoptotic and pro-angiogenic genes including bcl-xL, MCL1, c-FLIP-L, and VEGF [27].

These findings provide important clues to the role of protective bile acids in CRC and set the stage for exploring the centrality of the FXR signaling axis. Yang et al.‘s study, building on this background, demonstrated that altered TUDCA/GUDCA levels after cholecystectomy influence CRC development via FXR [19].

#### Context-dependent role of TUDCA/GUDCA

It is important to clarify that the terms “protective” and “pro-carcinogenic” for TUDCA/GUDCA are context-dependent. Under normal physiological conditions, exogenous TUDCA (as a pharmacological agent) exhibits cytoprotective, anti-apoptotic, and ER stress-relieving properties, which has led to its classification as a “protective” bile acid. However, after cholecystectomy, the continuous non-prandial bile flow creates a distinct biochemical milieu: elevated concentrations of TUDCA/GUDCA, together with increased secondary bile acids (DCA, LCA), collectively alter the bile acid pool composition and ratio. In this specific context, TUDCA/GUDCA may act as competitive antagonists at the FXR ligand-binding domain, especially when present in high concentrations relative to FXR agonists (e.g., CDCA). Thus, the same molecule can be protective when administered acutely but contributes to FXR suppression when chronically elevated in the post-cholecystectomy state. This duality is not a contradiction but rather a reflection of concentration-, ratio-, and context-dependent signaling.

#### The TUDCA biochemical paradox: from cytoprotector to FXR antagonist

A critical point of debate is the apparent contradiction between TUDCA’s well-established cytoprotective and anti-apoptotic properties and its proposed role as an FXR-suppressing agent in the post-cholecystectomy state. Under normal physiological conditions, TUDCA functions as a chemical chaperone, reducing ER stress and inhibiting mitochondrial permeability transition. However, the bile acid milieu after cholecystectomy is characterized by continuous flux and altered conjugation ratios. Recent structural studies suggest that TUDCA may act as a competitive antagonist at the FXR ligand-binding domain when present in high concentrations or in specific ratios with other bile acids (e.g., elevated DCA/LCA). Specifically, TUDCA has a lower binding affinity than CDCA but can outcompete weaker agonists under conditions of altered enterohepatic circulation. Moreover, TUDCA’s effect on FXR is context-dependent: in the presence of pro-inflammatory cytokines (e.g., TNF-α, IL-6), which are elevated after surgery, TUDCA may shift from a neutral to an inhibitory modulator. Thus, the paradoxical role of TUDCA reflects a concentration- and context-driven switch, not an inherent contradiction. This nuance is essential for understanding why TUDCA elevation after cholecystectomy correlates with FXR suppression and CRC promotion, whereas exogenous TUDCA supplementation in healthy models may be protective.

### The vicious cycle of microbiota–bile acid interactions

#### Bridging the latency gap: a multi-hit model

A major conceptual gap exists between the rapid onset of gut dysbiosis (weeks after cholecystectomy) and the clinical latency of CRC (5–15 years). We propose a multi-hit model wherein early microbiota shifts create a chronically altered bile acid landscape, but neoplastic transformation requires cumulative thresholds of DNA damage and clonal expansion. Specifically, elevated secondary bile acids (DCA, LCA) induce oxidative DNA damage (e.g., 8-OHdG formation) within months, yet most damaged cells undergo apoptosis. Only after repeated cycles of injury and regeneration—over years—do somatic mutations in key driver genes (e.g., APC, KRAS, TP53) accumulate. Additionally, secondary bile acids promote the expansion of pre-existing mutant clones by creating a selective growth advantage. Thus, the latency period reflects the time needed for: (1) accumulation of sufficient DNA damage burden, (2) clonal evolution of field cancerization, and (3) immune evasion. This model aligns with the observed site-specificity (proximal colon has longer mucosal exposure) and the protective effect of dietary fiber (which dilutes bile acids).

After cholecystectomy, gut microbiota changes and bile acid disturbances form a self-reinforcing vicious cycle that promotes CRC.

On one hand, altered bile acid composition reshapes the gut microbiota. High concentrations of secondary bile acids exert antimicrobial effects, inhibiting beneficial bacteria (e.g., *Bifidobacterium*, *Lactobacillus*) while selecting for bile-resistant potential pathogens (e.g., *Bacteroides*, *Desulfovibrio*). This selective pressure is a major driver of post-cholecystectomy dysbiosis.

On the other hand, the altered microbiota feeds back to modulate bile acid metabolism. Specific bacteria with 7α-dehydroxylase activity convert more primary bile acids into pro-carcinogenic secondary bile acids. Yang et al. confirmed that cholecystectomy increases *Ruminococcus gnavus* and decreases *Bifidobacterium breve*, promoting TUDCA/GUDCA production and thereby influencing CRC [19].

Dietary factors further modulate these interactions. High-fat diets exacerbate post-cholecystectomy intestinal inflammation and dysbiosis, suggesting that dietary intervention could be a potential strategy to interrupt this vicious cycle.

### Summary

Cholecystectomy-induced bile acid disturbances have a dual nature: increased production of pro-carcinogenic secondary bile acids (DCA, LCA) that promote CRC through DNA damage, oncogenic signaling, and apoptosis inhibition, along with altered levels of protective bile acids (UDCA, TUDCA) that weaken the gut’s natural defenses. This imbalance, coupled with gut microbiota dysbiosis, creates a vicious cycle driving colorectal carcinogenesis. The critical question of how these changes converge on specific molecular pathways, particularly the FXR signaling axis, will be addressed in the next section.

## Breakthrough: the central role of the FXR signaling axis

Farnesoid X receptor (FXR), a nuclear receptor activated by bile acids, has garnered increasing attention in CRC research. The 2025 study by Yang et al. in Nature Communications revealed for the first time the central role of the microbiota–bile acid–FXR axis in cholecystectomy-associated CRC, advancing the field from descriptive epidemiology to mechanistic understanding [19].

### Overview of FXR

#### Structure and function

FXR (encoded by NR1H4) is a bile acid-activated nuclear receptor highly expressed in the liver and intestine. In the intestine, FXR is most abundant in the terminal ileum and proximal colon, where it regulates bile acid homeostasis and barrier function [28]. As a master regulator of bile acid homeostasis, FXR not only controls bile acid synthesis, transport, and metabolism but also participates in maintaining intestinal homeostasis, protecting the gut barrier, and suppressing inflammation.

#### Tumor-suppressive role in CRC

Multiple studies have shown that FXR expression is downregulated in CRC tissues and that low FXR expression correlates with poor prognosis. Mechanistically, FXR exerts tumor-suppressive effects by antagonizing Wnt/β-catenin signaling. Specifically, FXR directly interacts with β-catenin, disrupting the formation of β-catenin/TCF4 complexes and thereby inhibiting transcription of downstream pro-carcinogenic target genes such as c-Myc and cyclin D1. Additionally, FXR activation can suppress JAK2/STAT3 signaling via upregulation of SOCS3 [29].

#### Bidirectional relationship with gut microbiota

FXR and the gut microbiota engage in bidirectional crosstalk. The microbiota influences FXR activity by metabolizing bile acids, while FXR signaling, in turn, shapes microbial composition by modulating the bile acid pool and regulating antimicrobial peptide expression [28]. FXR expression progressively declines during the adenoma–carcinoma sequence [30]. Furthermore, antagonism of intestinal FXR has been shown to induce proliferation and DNA damage in Lgr5 + stem cells, highlighting FXR’s role in maintaining stem cell homeostasis [31].

### Key findings from Yang et al. (*Nature Communications*, 2025, in Table 2)

Yang and colleagues provided the first comprehensive molecular dissection of how cholecystectomy-associated gut microbiota dysbiosis promotes colorectal tumorigenesis. Using animal models, clinical cohorts, multi-omics, and functional experiments, they delineated a complete “microbiota–bile acid–FXR” axis [19].

**Table 2:** Summary of key experimental findings from Yang et al. (Nat Commun 2025)

Study Component	Key Findings	Experimental Approaches
Microbial changes	↓ *B. breve*, ↑ *R. gnavus*	Metagenomic sequencing, qPCR validation
Bile acid changes	↑ GUDCA (human), ↑ TUDCA (mouse)	Targeted metabolomics
Molecular mechanism	FXR suppression, disruption of FXR/β-catenin interaction	RNA-seq, co-immunoprecipitation
Causal validation	Microbiota drives tumorigenesis	FMT, mono-colonization, antibiotic depletion
Therapeutic intervention	OCA prevents tumors	Animal model intervention

This table summarizes key experimental findings from Yang et al. (Nat Commun 2025), which elucidated the microbiota-bile acid-FXR axis in cholecystectomy-associated colorectal carcinogenesis using multi-omics approaches and functional validation experiments

*B. breve* *Bifidobacterium breve*, *R. gnavus* *Ruminococcus gnavus*, *GUDCA* glycoursodeoxycholic acid, *TUDCA* tauroursodeoxycholic acid, *FXR* farnesoid X receptor, *FMT* fecal microbiota transplantation, *OCA* obeticholic acid

#### Microbial changes: decreased *Bifidobacterium breve*, increased *Ruminococcus gnavus*

Metagenomic sequencing of fecal samples from cholecystectomized patients and healthy controls revealed significant alterations, with a marked decrease in *Bifidobacterium breve* and increase in *Ruminococcus gnavus*. These changes were validated in both humans and mice, suggesting they may serve as core microbial signatures of cholecystectomy.

#### Bile acid changes: elevated GUDCA in humans and TUDCA in mice

Targeted metabolomics showed increased levels of GUDCA in humans and TUDCA in mice after cholecystectomy. TUDCA/GUDCA, conjugated forms of the protective bile acid UDCA, directly influence intestinal carcinogenic susceptibility.

#### Molecular mechanism: FXR suppression and disruption of FXR/β-catenin interaction

RNA sequencing and co-immunoprecipitation revealed that altered bile acid profiles after cholecystectomy lead to FXR signaling suppression and disruption of the FXR/β-catenin interaction. This disruption releases β-catenin from the complex, allowing its nuclear translocation and activation of downstream pro-carcinogenic target genes, ultimately accelerating colorectal tumorigenesis.

#### Causal validation via fecal microbiota transplantation and mono-colonization

##### Fecal Microbiota Transplantation (FMT)

Transfer of feces from cholecystectomized patients to mice significantly increased colonic tumor burden and induced more advanced pathology in recipient animals.

##### Mono-colonization

Mono-colonization with *R. gnavus* promoted TUDCA production and colorectal tumorigenesis, while supplementation with *B. breve* reversed these effects.

##### Antibiotic depletion

Antibiotic-mediated gut microbiota depletion abolished the pro-carcinogenic effect of cholecystectomy, confirming its microbiota dependence.

#### Intervention evidence: preventive effect of the FXR agonist Obeticholic Acid (OCA)

Strikingly, treatment with the FXR agonist obeticholic acid (OCA) effectively prevented cholecystectomy-associated colorectal tumors in mice. This finding not only identifies a novel therapeutic target but also opens the possibility of repurposing OCA, already FDA-approved for primary biliary cholangitis, for cancer prevention.

### Supporting evidence from other FXR-related studies

Yang et al.‘s findings are complemented by other recent studies highlighting the centrality of FXR signaling in CRC.

#### 3-oxo-LCA as an FXR agonist inhibiting CRC

Sun et al. (2025, Cancer Research) discovered that the microbial bile acid metabolite 3-oxolithocholic acid (3-oxo-LCA) acts as a potent FXR agonist, restoring FXR activity in vitro and in vivo [32]. In APC^(min/+) mice, 3-oxo-LCA treatment reduced bile acid levels, enhanced intestinal barrier function, decreased tumor burden, and inhibited tumor initiation. Mechanistically, 3-oxo-LCA suppressed intestinal stem cell proliferation and promoted apoptosis via FXR activation. This study complements Yang et al.‘s work by demonstrating an alternative route to FXR activation (direct agonism vs. modulation of endogenous ligands).

#### *Weissella cibaria* probiotic suppresses colitis-associated CRC via the microbiota–bile acid–FXR axis

Hao et al. (2025, mSystems) showed that oral administration of *Weissella cibaria* in a mouse model of colitis-associated CRC reshaped the gut microbiota, reduced bile salt hydrolase activity and unconjugated bile acid levels, upregulated intestinal FXR expression, inhibited NF-κB signaling, and significantly attenuated tumor burden [33]. This provides proof-of-concept that probiotic intervention targeting the FXR axis is feasible.

#### Multiple anti-CRC mechanisms of FXR agonists

Earlier studies have also demonstrated that OCA exerts anti-CRC effects through diverse mechanisms. Yu et al. [20] reported that OCA inhibited proliferation, invasion, cell cycle progression, and induced apoptosis in colon cancer cells via SOCS3-mediated JAK2/STAT3 pathway suppression, further supporting the rationale for OCA in CRC prevention [34].

### Summary

**Fig. 2:**
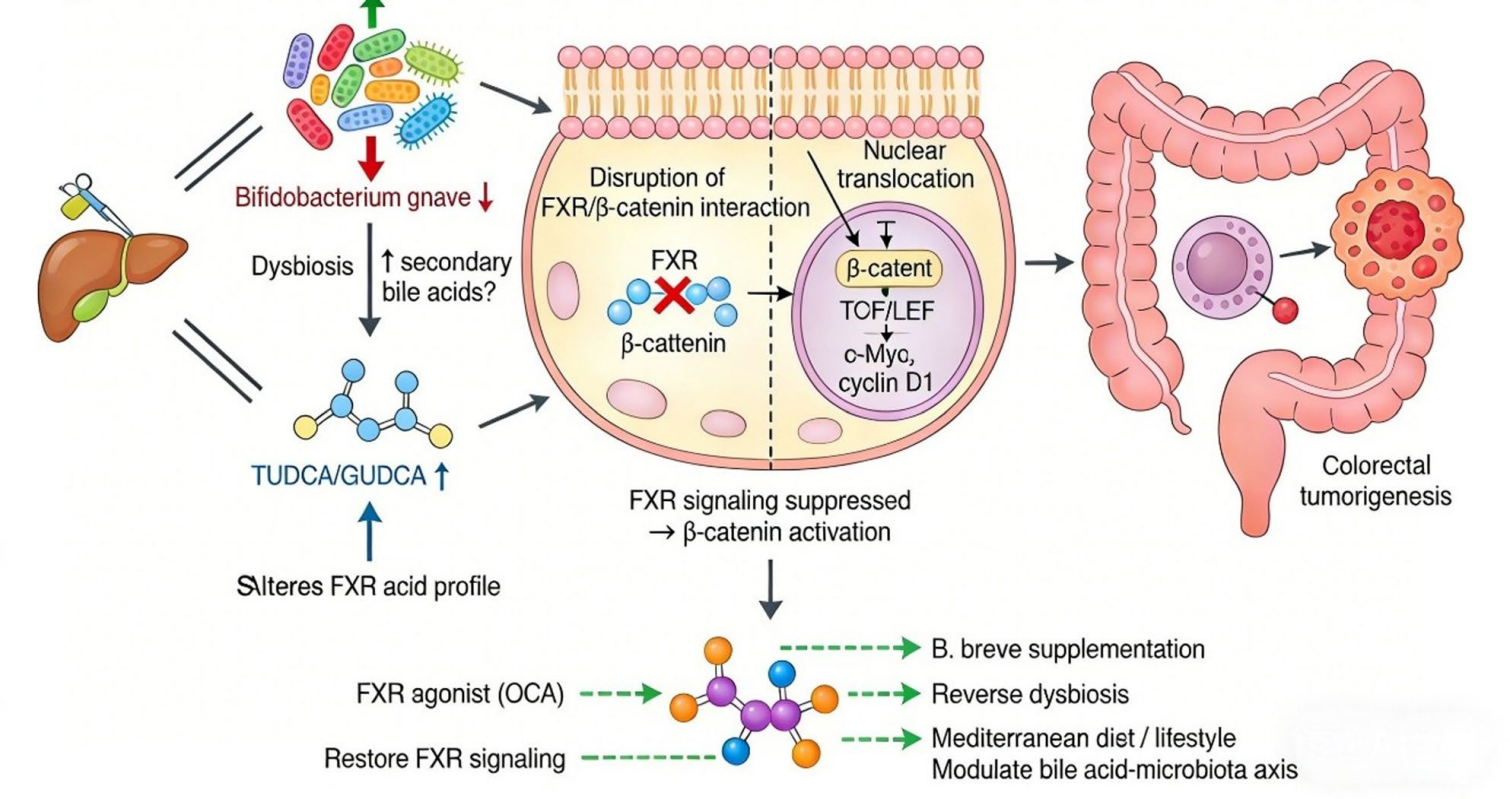
Schematic illustration of the microbiota-bile acid-FXR axis in cholecystectomy-associated colorectal carcinogenesis. Cholecystectomy leads to continuous bile flow into the intestine, resulting in gut microbiota dysbiosis characterized by decreased *Bifidobacterium breve* and increased *Ruminococcus gnavus*. These microbial shifts alter the bile acid profile, elevating levels of TUDCA/GUDCA. The altered bile acids suppress farnesoid X receptor (FXR) signaling and disrupt the FXR/β-catenin interaction, leading to β-catenin nuclear translocation and activation of pro-carcinogenic target genes (e.g., c-Myc, cyclin D1; encoded by MYC and CCND1), ultimately promoting colorectal tumorigenesis. Potential intervention strategies are indicated with dashed arrows: FXR agonists (obeticholic acid, OCA) restore FXR signaling; *B. breve* supplementation reverses dysbiosis; dietary interventions (e.g., Mediterranean diet) modulate the bile acid-microbiota axis

The FXR signaling axis is a central hub in cholecystectomy-associated CRC (Fig. 2). Yang et al.‘s landmark study systematically delineated the complete pathway: cholecystectomy → microbial shifts (↓ *B. breve*, ↑ *R. gnavus*) → altered bile acid metabolism (↑ TUDCA/GUDCA) → FXR suppression → disruption of FXR/β-catenin interaction → β-catenin activation → tumorigenesis [19]. The downstream transcriptional targets of β-catenin, including CCND1 and MYC, are highlighted in Fig. 1B. Complementary studies on 3-oxo-LCA [32] and *W. cibaria* [33] reinforce the centrality of this axis from different angles. These mechanistic insights not only explain the epidemiological observations of proximal predominance and sex differences but also provide clear targets for clinical intervention (FXR agonists, specific probiotics).

## Potential intervention strategies and clinical translation

Given the central role of the microbiota–bile acid–FXR axis in cholecystectomy-associated CRC, interventions targeting this axis hold significant promise for clinical translation. Strategies range from repurposing approved drugs to probiotics and dietary modifications, offering multiple levels of risk reduction.

### FXR agonists: preventive potential of Obeticholic Acid (OCA)

The most direct intervention evidence comes from Yang et al., showing that the FXR agonist OCA effectively prevents cholecystectomy-associated colorectal tumors in mice [19]. This finding has substantial translational potential, as OCA is already FDA-approved for primary biliary cholangitis, with well-characterized safety and pharmacokinetics.

#### Mechanism of action

OCA, a selective FXR agonist, restores FXR signaling suppressed by cholecystectomy, thereby repairing the FXR/β-catenin interaction and blocking β-catenin nuclear translocation and downstream pro-carcinogenic gene expression. In animal studies, OCA significantly reduced colorectal tumor burden in cholecystectomized mice [19].

#### Preclinical support

Beyond Yang et al.‘s work, earlier studies have demonstrated OCA’s anti-CRC effects via multiple mechanisms. Yu et al. [20] showed that OCA inhibits colon cancer cell proliferation, invasion, and induces apoptosis through SOCS3-mediated JAK2/STAT3 pathway suppression [34]. Sun et al. [31] further confirmed that the microbial metabolite 3-oxo-LCA, an FXR agonist, reduces tumor burden in APC^(min/+) mice [32].

#### Clinical translation prospects

As an approved drug, OCA’s repurposing pathway is relatively straightforward. Future prospective clinical trials are needed to validate its efficacy in preventing CRC in cholecystectomized populations, determine optimal dosing and duration, and identify subgroups most likely to benefit. However, long-term safety data require further accumulation, especially in non-cirrhotic populations. OCA’s use in liver disease has revealed dose-dependent adverse effects, including pruritus (occurring in up to 60% of patients), LDL cholesterol elevation (10–20%), increased gallstone risk, and potential hepatotoxicity [35, 36]. These safety concerns must be carefully considered in preventive applications.

#### Clinical feasibility and alternative approaches

While OCA demonstrates efficacy in preclinical CRC prevention, its systemic adverse effects—particularly dose-dependent pruritus (up to 60% of patients) and LDL cholesterol elevation (10–20%)—pose major obstacles for primary prevention in millions of healthy cholecystectomized individuals. Therefore, more clinically viable strategies include gut-restricted FXR agonists (e.g., fexaramine or non-absorbable synthetic agonists) that activate intestinal FXR without systemic exposure. Such agents could restore local FXR signaling in the colon while avoiding hepatic and systemic side effects. Alternatively, modulation of the gut microbiota to enhance endogenous FXR ligands (e.g., 3-oxo-LCA) or dietary interventions that reshape the bile acid pool may offer safer, long-term preventive approaches. These strategies should be prioritized in future clinical trials.

### Probiotic/prebiotic interventions

Given the specific microbial alterations after cholecystectomy, supplementation with targeted probiotics or prebiotics represents another promising strategy.

#### Bifidobacterium breve supplementation

Yang et al. found that *B. breve* is significantly reduced after cholecystectomy [19]. In animal experiments, *B. breve* supplementation reversed the pro-carcinogenic effects of cholecystectomy, highlighting its potential as an intervention target. This aligns with earlier evidence that Lactobacillus and Bifidobacterium intake can suppress tumors by modulating gut microbiota.

#### Weissella cibaria probiotic

Hao et al. [32] demonstrated that *W. cibaria* suppressed colitis-associated CRC in mice by reshaping the gut microbiota, reducing bile salt hydrolase activity and unconjugated bile acid levels, upregulating intestinal FXR expression, and inhibiting NF-κB signaling [33].

#### Prebiotics and dietary fiber

Systematic reviews indicate that supplementation with oligosaccharides or dietary fiber increases butyrate-producing bacteria, thereby inhibiting tumorigenesis [37]. Butyrate, a key short-chain fatty acid, serves as the primary energy source for colonocytes and exerts anti-inflammatory, barrier-protective, and anti-proliferative effects. For cholecystectomized patients, fiber-rich diets may confer protection by promoting butyrate producers.

#### Multi-strain synbiotics

Given the complex microbial shifts involving multiple genera, single-strain supplementation may be insufficient to fully restore homeostasis. Future research should explore synbiotic combinations containing *B. breve*, lactobacilli, and other beneficial strains along with prebiotics.

### Dietary interventions: from high-fat warnings to mediterranean diet protection

Diet is a powerful environmental modulator of the microbiota–bile acid axis and occupies a central role in CRC prevention after cholecystectomy.

#### Risk amplification by high-fat diet

Epidemiological evidence suggests that high-fat diets exacerbate post-cholecystectomy intestinal inflammation and dysbiosis. Mechanistically, high-fat diets increase bile acid secretion, providing more substrate for pro-carcinogenic secondary bile acid production. They also directly alter gut microbial composition, favoring bile-resistant potential pathogens [38]. Therefore, limiting high-fat intake may be a fundamental risk-reduction measure for cholecystectomized individuals.

#### Protective potential of the Mediterranean diet

An ongoing RCT funded by the U.S. National Cancer Institute is systematically evaluating the effects of a Mediterranean diet combined with weight loss on the bile acid–microbiota axis in CRC prevention, focusing on African Americans, a high-risk population [39]. The core hypothesis is that a Mediterranean diet (rich in plant-based foods, fiber, and healthy fats) can shift bile acid profiles, suppress pro-carcinogenic bacteria producing hydrogen sulfide and DCA, and thereby lower CRC risk. This study design offers valuable insights for dietary interventions in cholecystectomized patients.

#### Specific dietary recommendations

##### Increase dietary fiber intake

Whole grains, legumes, vegetables, and fruits promote butyrate-producing bacteria and enhance SCFA production.

##### Limit saturated fat and animal protein

Red and processed meats should be consumed in moderation to avoid chronic high-fat intake.

##### Adopt a Mediterranean diet pattern

Emphasize olive oil as the primary fat source, with ample fish, nuts, vegetables, and whole grains.

##### Maintain healthy weight

Obesity is an independent CRC risk factor; weight loss interventions may provide dual benefits by improving metabolism and microbiota.

### High-risk population screening and surveillance

Based on epidemiological risk patterns, enhanced CRC screening for specific cholecystectomized subgroups is clinically warranted.

#### High-risk population definition

##### Female sex

Multiple studies indicate a more pronounced risk increase in women, particularly for proximal colon cancer [20].

##### Proximal colon location

The most consistent finding is elevated risk of proximal colon cancer; screening should prioritize this segment [12, 15].

##### Postoperative window of 5–15 years

This is the critical period when biological effects manifest, warranting intensified surveillance [15].

##### Concomitant high-risk factors

Smoking, obesity, high-fat diet, and family history of CRC may synergize with cholecystectomy.

##### Younger age (< 50 years)

Some studies suggest increased risk of early-onset CRC [8].

#### Screening strategy recommendations

##### Baseline screening

A high-quality colonoscopy before or within one year after cholecystectomy to exclude pre-existing asymptomatic neoplasia.

##### Long-term follow-up

For high-risk individuals, enhanced surveillance starting 5 years post-surgery, with colonoscopy every 3–5 years (interval tailored to individual risk). Low-risk individuals may follow general population guidelines.

##### Focus on the proximal colon

During colonoscopy, special attention should be paid to the proximal colon; chromoendoscopy or high-definition imaging may be considered to improve lesion detection.

##### Clinical decision support

A 2024 study in the Journal of Gastrointestinal Surgery addressed management of patients with asymptomatic gallstones undergoing CRC surgery. It found that concomitant prophylactic cholecystectomy during elective CRC resection did not increase postoperative complications or mortality but significantly reduced biliary complications (26.5% vs. 0.0%; *p* < 0.01). This suggests that for CRC patients with asymptomatic gallstones, concurrent cholecystectomy may be the preferred strategy, especially in those aged ≥ 65 years, women, and those with multiple small stones [40].

## Conclusion and future directions

### Summary: from epidemiological controversy to mechanistic breakthrough

Over four decades, research on cholecystectomy and CRC risk has evolved from conflicting epidemiological reports to a coherent mechanistic understanding. Cholecystectomy, one of the most common surgeries worldwide (affecting ~ 5% of the population), has been linked to CRC in some but not all studies. However, refined analyses have revealed consistent patterns: site-specificity (proximal colon cancer predominance), sex differences (higher risk in women), and temporal patterns (peak incidence 5–15 years post-surgery). Large cohort studies have demonstrated a significantly increased risk of proximal colon cancer after cholecystectomy, with adjusted odds ratios reaching up to 2.42 (95% CI 1.85–3.15) [15], while a Korean population-based study reported a modest increase in overall CRC risk (HR 1.15, 95% CI 1.06–1.25) [14]. A 2025 systematic review in *Cancers* encompassing 21 cohort studies confirmed that cholecystectomy is associated with an elevated risk of proximal colon cancer, with several studies showing consistent positive associations [12]. Furthermore, a meta-analysis of 14 cohort studies reported a 39% increased risk of colorectal adenoma after cholecystectomy (OR 1.39, 95% CI 1.15–1.68), with a stronger effect observed in East Asian populations (OR 1.95) [20].

Mechanistically, the field has advanced from descriptive observations to molecular dissection. Yang et al.‘s 2025 Nature Communications study delineated the complete pathway: cholecystectomy → microbial shifts (↓ *B. breve*, ↑ *R. gnavus*) → altered bile acid metabolism (↑ TUDCA/GUDCA) → FXR suppression → disruption of FXR/β-catenin interaction → β-catenin activation → tumorigenesis [19]. This discovery not only explains the proximal predominance (greater bile acid exposure) but also establishes FXR as a central hub. Complementary studies on 3-oxo-LCA [32] and *W. cibaria* [33] reinforce the centrality of the FXR axis from different perspectives.

In essence, this review establishes that cholecystectomy-associated CRC risk is real, site-specific, and mechanistically driven by the microbiota-bile acid-FXR axis, with actionable implications for surveillance and prevention.

### Outstanding questions

Despite these advances, several critical questions remain.

#### Prospective validation of causality

Current evidence is largely from retrospective cohorts. Although methods like propensity score matching have minimized confounding, definitive causal inference requires prospective randomized controlled trials or Mendelian randomization studies. Rigorous prospective studies assessing dose–response and temporal relationships are lacking.

#### Mechanisms underlying population differences

Epidemiological data reveal significant variations by race, sex, and age. A meta-analysis has demonstrated that East Asians have a nearly twofold higher risk than North Americans (OR 1.95 vs. 1.16) [20]. Young adults (18–45 years) appear to face a disproportionately higher risk following cholecystectomy, as suggested by studies on early-onset colorectal cancer [8], although precise estimates specific to cholecystectomy require further investigation. Sex-specific effect sizes also differ, with women facing higher risk for proximal colon cancer [20]. The biological basis for these disparities remains unclear but may involve interactions between genetic background, baseline microbiota, and dietary habits.

#### Clinical safety and tissue specificity of FXR agonists

While OCA shows promise in preclinical CRC prevention, its clinical translation faces challenges. In liver disease patients, OCA has been associated with dose-dependent pruritus, LDL cholesterol elevation, increased gallstone risk, and potential hepatotoxicity [35, 36]. Moreover, FXR exhibits tissue-specific functions [29], and strategies to selectively activate intestinal FXR for anti-tumor effects while avoiding hepatic adverse effects need to be developed.

#### Dynamic regulation of the microbiota–bile acid–FXR network

Most studies provide snapshot analyses at single time points. The dynamic evolution of this network after cholecystectomy, the formation of a vicious cycle between dysbiosis and altered bile acids, and the downstream effects of FXR suppression remain poorly understood. Answering these questions will require longitudinal multi-omics and dynamic network modeling [22].

### Outlook: personalized screening and intervention from a precision medicine perspective

Emerging evidence is paving the way for personalized risk stratification and intervention in cholecystectomized populations [12].

#### Precision risk prediction

Integrative models combining epidemiological risk factors and biomarkers can identify high-risk individuals. Key dimensions include:

#### Demographics

young age (< 50 years), female sex, East Asian ethnicity.

#### Clinical features

proximal colon cancer, postoperative window of 5–15 years.

#### Microbial signatures

decreased *B. breve*, increased *R. gnavus*.

#### Metabolic markers

TUDCA/GUDCA levels, FXR activity.

#### Genetic susceptibility

polymorphisms in FXR and bile acid metabolism genes.

#### Stratified screening

Based on risk stratification, personalized screening protocols can be implemented. For high-risk individuals, enhanced surveillance starting 5 years post-surgery with colonoscopy every 3–5 years, focusing on the proximal colon. Low-risk individuals may follow general population guidelines. This approach aligns with recommendations in recent systematic reviews that emphasize personalized surveillance strategies incorporating gallbladder history, especially for high-risk populations [12, 18].

#### Multi-level intervention strategies

##### Pharmacological

FXR agonists like OCA, already approved, offer a repurposing opportunity. Prospective trials are needed to evaluate efficacy, optimal dosing, and long-term safety in cholecystectomized populations.

##### Microbiota-targeted

Supplementation with *B. breve*, *W. cibaria*, or other probiotics, and prebiotics to promote butyrate producers, aiming to restore microbial homeostasis.

##### Dietary

Adoption of a Mediterranean diet, increased fiber intake, and restriction of high-fat foods to modulate the bile acid–microbiota axis.

##### Lifestyle

Maintenance of healthy weight, smoking cessation, and moderation of alcohol intake to mitigate synergistic carcinogenic effects.

##### Multi-center collaboration and data sharing

Fully elucidating the relationship between cholecystectomy and CRC requires large-scale prospective cohorts with long-term follow-up. Data sharing and standardized study designs will enable validation of prediction models, identification of effect modifiers, and optimization of intervention strategies.

From basic research to clinical translation, and from population-wide screening to personalized prevention, the management of CRC risk after cholecystectomy is advancing toward precision medicine. These developments will not only provide scientific guidance for the hundreds of millions of cholecystectomized individuals worldwide but also serve as a paradigm for leveraging the “microbiota–metabolism–host” axis in cancer prevention.

However, the stability of TUDCA/GUDCA ratio as a clinical biomarker requires validation. Preliminary evidence suggests that this ratio is relatively stable intra-individually over months, but can be influenced by acute dietary fat intake and concomitant medications (e.g., statins). Therefore, repeated measurements or standardized sampling conditions (e.g., fasting) are recommended before implementing the proposed personalized framework.

## Data Availability

No datasets were generated or analysed during the current study.
